# A Novel Systematic Error Compensation Algorithm Based on Least Squares Support Vector Regression for Star Sensor Image Centroid Estimation

**DOI:** 10.3390/s110807341

**Published:** 2011-07-25

**Authors:** Jun Yang, Bin Liang, Tao Zhang, Jingyan Song

**Affiliations:** Department of Automation, Tsinghua University, Beijing 100084, China; E-Mails: jun-yang07@mails.tsinghua.edu.cn (J.Y.); bliang@tsinghua.edu.cn (B.L.); jysong@tsinghua.edu.cn (J.S.)

**Keywords:** star sensor, subpixel, centroid estimation, systematic error compensation, LSSVR

## Abstract

The star centroid estimation is the most important operation, which directly affects the precision of attitude determination for star sensors. This paper presents a theoretical study of the systematic error introduced by the star centroid estimation algorithm. The systematic error is analyzed through a frequency domain approach and numerical simulations. It is shown that the systematic error consists of the approximation error and truncation error which resulted from the discretization approximation and sampling window limitations, respectively. A criterion for choosing the size of the sampling window to reduce the truncation error is given in this paper. The systematic error can be evaluated as a function of the actual star centroid positions under different Gaussian widths of star intensity distribution. In order to eliminate the systematic error, a novel compensation algorithm based on the least squares support vector regression (LSSVR) with Radial Basis Function (RBF) kernel is proposed. Simulation results show that when the compensation algorithm is applied to the 5-pixel star sampling window, the accuracy of star centroid estimation is improved from 0.06 to 6 × 10^−5^ pixels.

## 1. Introduction

The star tracker is a satellite-based embedded system which estimates the orientation of the satellite in space. This information is essential for any space mission, as it supplies all attitude data required for satellite control. There are other sensors used for the same purpose (gyroscope, sun tracker, magnetometer, GPS), but star trackers are more accurate and allow for attitude estimation without prior information [1]. For these reasons star trackers are used onboard 3-axis stabilized spacecraft. Star trackers estimate the orientation directly from the images of stars taken by an onboard camera. The estimation is based on a comparison of the star locations in the image with those in the predefined catalogue. One important factor influenced the performance of the star tracker is the star centroid location estimation in the image. This process becomes difficult when noise exists. This work applies the Least Square Support Vector Regression (LSSVR) with Radial Basis Function (RBF) kernel to improve the estimation process.

The noise influence on the estimation process can be divided into two types, the random noise and the systematical noise. The random noise includes the short noise, dark current noise, CCD readout noise, and radiation noise, which are closely related with the hardware of the CCD sensor [2]. In order to obtain high accuracy star locations in the image, sub-pixel centroid algorithms should be adopted, namely, the center of mass (COM), polynomial and B-spline interpolators [3]. The systematic noise is due to the nature of the centroid algorithm. The systematic noise of the centroid algorithm can cause several arc-seconds accuracy loss, so it is essential to analyze the systematic error and design a compensation method to improve the accuracy of star centroid location estimation in the image. In this paper, the systematic error is discussed in detail and the random noise will be only briefly analyzed.

The properties of the systematic error have been investigated by many scholars. In general, systematic error of centroid estimation is related with the energy distribution of starlight on star image (Gaussian width), the frequency of sampling, the size of sampling window and the actual position of star point. Grossman *et al.* [4] pointed out that the systematic error was reduced by increasing degrees of blur and by the wider defocusing of the neighbor pixels of the starlight. However, Hegedus *et al.* [5] pointed out that the error firstly decreases and then increases as star Gaussian width is increased. Stanton *et al.* [6] obtained a roughly sinusoid functional relationship between systematic error and the actual position of star point under fixed blur size. Alexander *et al.* [7] analyzed the systematic error through a spatial-frequency-based approach caused by the center of mass algorithm. Jean [8] supplemented Alexander’s work and proposed a Fourier phase shift method to calculate the sub-pixel position under more complex signals. Rufino *et al.* [9] obtained the starlight intensity distribution point spread function (PSF) considering diffraction and CCD defocus, and used the BP neural network method to compensate the systematic error. JIA *et al.* [10] studied the systematic error utilizing a frequency domain method considering sampling frequency limitation and sampling window limitation. He also proposed an analytical compensation algorithm to reduce the systematic error of star centroid estimation.

This paper analyzes the systematic error caused by the center of mass (COM) centroid estimation algorithm. Through the frequency domain approach analysis and numerical simulations, it is found that the systematic error consists of an approximation error and a truncation error. The approximation error results from the discretization approximation, which is caused when the spacial frequency of a star image is higher than the sampling frequency of the detector. The truncation error will appear when the size of the sampling window is smaller than the Gaussian width of the star intensity distribution. A criterion for choosing the size of the sampling window is given to reduce the truncation error as much as possible. Through numerical simulations, the systematic error can be evaluated as a function of the actual star centroid positions under different Gaussian widths of the star intensity distribution. In order to eliminate the systematic error, a novel systematic error compensation algorithm based on the least squares support vector regression (LSSVR) with Radial Basis Function (RBF) kernel is proposed. This novel algorithm can control the function estimation kernel shape and prediction accuracy. The experimental results demonstrate that the proposed approach can improve the accuracy of the star centroid position estimation dramatically.

The rest of this paper is organized as follows. In Section 2, the error of star centroid estimation algorithm is analyzed from three aspects through a frequency domain approach and numerical simulations: the integral error, the approximation error and the truncation error. A detailed description of our novel compensation algorithm based on the LSSVR is given in Section 3. In Section 4, the performance of the LSSVR compensation algorithm is evaluated. Finally, the conclusions of the paper are drawn in Section 5.

## 2. Error Analysis of Star Centroid Estimation Algorithm

It is well known that the star centroid calculation is used to pinpoint location. In order to adopt digital centroid algorithms to achieve sub-pixel accuracy in star centroid position estimation, the star sensor camera should be defocused slightly in order to spread the star energy over several neighboring pixels [11]. The center of mass (COM) algorithm is the most widely method used to calculate the centroid position of star images, and the error analysis is based on the COM algorithm [1,2,4,10].

### 2.1. The Integral Error of Center of Mass (COM) Algorithm

It is evident that the sub-pixel accuracy star centriod cannot be obtained by one single pixel directly. The COM algorithm uses several neighbor pixels around the brightest pixel to calculate the sub-pixel star centroid position. The ideal star centroid position in the image plane is *x̂_c_* and *ŷ_c_*, which can be computed by:

(1) x^c^=∫∫WxI(x,y)dxdy∫∫WI(x,y)dxdy,              yc=∫∫WyI(x,y)dxdy∫∫WI(x,y)dxdy

where *W* is the sampling window area that include all validated neighbor pixels around the starlight in the image plane, *x* and *y* are the coordinates of the pixels in *W*, *I*(*x, y*) is the detected signal irradiance intensity at pixel (*x, y*). Equation (1) is the COM algorithm’s theory model, it should be discretized when it used in digital computation. After the discretization, Equation (1) can be written as:

(2) x^g^=∑i=1nxiIi∑i=1nIi,          yg=∑i=1nyiIi∑i=1nIi

where *x̂_g_* and *ŷ_g_* are the actual star centroid position in the image plane after discretization. *W* in Equation (1) replaces the discrete *n* pixels to constitute the sampling window, *x_i_* and *y_i_* are the coordinates of the geometric center of the *i-th* pixel, *I_i_* and is the irradiance intensity integration of the *i-th* pixel.

From Equation (2), it can be found that there are three factors can influence the star centroid estimation accuracy: the size of sampling window *W*, the *i-th* pixel coordinates *x_i_* in *W* and the signal intensity *I_i_* in corresponding pixels. The systematic error is caused by the discrete approximation of the coordinate *x_i_* and truncating the sampling window *W*, and the uncertainty in detecting *I_i_* leads to random noise. The 1-D situation in the x direction will be discussed, and the analysis is also valid for both the x and y direction in 2-D situation. Assuming the systematic error and the random noise are small and not correlated, then the integral error of the COM can be described by the expression [9]:

(3) σX˜g2=∑i=1n[(∂x^g^∂xi)2σx2+(∂xg∂Ii)2σI2]

where *σ_x̂_g__* is the integration error of *x̂_g_*, *σ_x_* is the systematic error resulting from the use of the pixel geometrical center to substitute the irradiance integration over a whole pixel and truncating the sampling window. *σ_I_* is the random error caused by various noises, namely, the short noise, dark current noise, CCD readout noise, and radiation noise *etc.*

Firstly, we consider random error which is caused by the uncertainty in detecting *I_i_*. We assume that the measured signal intensity *I_i_* at the pixel *x_i_* consists of two components: a ‘true’ intensity *E_i_*, and the noise intensity σ*_I_*, then the *I_i_* *= E_i_* + *σ_I_*. The derivatives in Equation (3) can be computed from Equation (2), and can be written as:

(4) $$\sigma_{{\tilde{X}}_{g},I}^{2}=\frac{{\left[\sum_{i=1}^{n}\left(x_{i}-x_{0}\right)\right]}^{2}\sigma_{I}^{2}}{I_{0}^{2}}={\left(\frac{\sigma_{I}}{I_{0}}\right)}^{2}\sum_{i=1}^{n}{\left[(x_{i}-x_{0})\right]}^{2}$$

where the total signal $I_{0}=\sum_{i=1}^{n}I_{i}$, the ‘true’ signal $E_{0}=\sum_{i=1}^{n}E_{i}$, and the ‘true’ star centroid position $x_{0}=\sum_{i=1}^{n}x_{i}E_{i}/E_{0}$.

If the *σ_I_* is small, the *I*_0_ ≈ *E*_0_, then through the Equation (4), we can find that the random error is inversely proportional to the signal to noise ratio (SNR). Enhancing the SNR can then reduce the random noise effectively. In this study, the random error analysis is not the key content. Many random noise elimination algorithms are described elsewhere [4,12] and are not covered in this paper.

In this paper, the analysis of systematic error is our main topic. From Equation (3), one also can use a derivative of the parameter *x_i_* to determine the systematic error, and this can be expressed as:

(5) $$\sigma_{{\tilde{X}}_{g},x}^{2}=\sigma_{x}{}^{2}\sum_{i=1}^{n}{\left(\frac{I_{i}}{I_{0}}\right)}^{2}$$

As we can see, the systematic error *σ_X̃_g_, x_* cannot be calculated directly through Equation (5), because there is little information about the *σ_x_* in time domain. In order to get more information to express the systematic error explicitly, we will analyze the systematic error using the frequency domain based method and numerical simulations.

### 2.2. Theoretical Analysis of the Systematic Approximation Error under Sampling Frequency Limitation

In this section, frequency domain analysis is adopted to get more information about the relationship between the systematic error and the ideal star centroid position just consideration of sampling frequency limitation. Under the condition of the spacial frequency of star image being higher than the sampling frequency of the detector, one type of systematic error named approximation error in calculating the star centroid position will be caused. We derive an approximate sinusoidal relationship between the approximation systematic error and the ideal star centroid position. The theoretical relationship function can inspire us to design some novel algorithms to compensate the systematic error.

**Figure 1.**
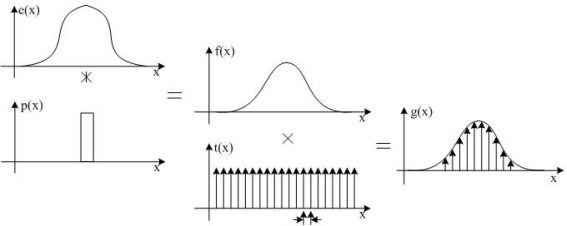
The process of star image sampling: e(x) is the starlight stripe intensity profile function; p(x) is the pixel sensitivity function; f(x) is the continuing pixel signal function; t(x) is the sampling function; g(x) is the discrete pixel signal function.

The star image sampling process is illustrated in Figure 1, and can be divided into two steps. The waveform e(x) is the intensity profile of the starlight stripe projected on the surface of the CCD. The signal intensity function e(x) is convoluted with the pixel sensitivity function p(x) to generate the continuing pixel signal function f(x). After multiplying the pixel sampling function t(x), we can get the discrete signal function g(x), which can be written as:

(6) $$\begin{array}{l} \begin{array}{ll} \begin{array}{l} f(x)=I(x,x_{0})⊗p(x)\\ g(x)=f(x)\times t(x) \end{array} & \Rightarrow g(x)=I(x,x_{0})⊗p(x)\times t(x) \end{array} \end{array}$$

When the CCD’s fill factor is approximated to 100%, the pixel sensitivity function p(x) is equal to a rectangle function. t(x) is the sampling function, its sampling frequency is *f_s_* = 1/*T* and is a comb function, T is the length of pixel. The p(x) and t(x) are given as follows:

(7) $$p(x)=\mathrm{rect}(x),\;\;t(x)=\mathrm{comb}(x)=\sum_{k=-\infty }^{k=\infty }\delta (x-\mathrm{kT})$$

The Fourier transform of the continuous function f(x) can be written as:

(8) $$F(s)=\int_{-\infty }^{\infty }f(x)\text{exp}(-2\pi \mathrm{isx})dx$$

and the derivative of the *F*(*s*) can be expressed *F*′(*s*) by as:

(9) $$F^{\prime }(s)=-2\pi i\int_{-\infty }^{\infty }\mathrm{xf}(x)\text{exp}(-2\pi \mathrm{isx})\mathrm{dx}$$

Then the ideal centroid position *x̂_c_* of f(x) can be calculated through Equations (8) and (9), as stated by Alexander [7]:

(10) $${\hat{x}}_{c}=\frac{\int_{-\infty }^{\infty }\mathrm{xf}(x)\mathrm{dx}}{\int_{-\infty }^{\infty }f(x)\mathrm{dx}}=-\frac{F^{\prime }(0)}{2\pi \mathrm{iF}(0)}$$

Likewise, the centroid of the sampled function *x̂_g_* can be written as:

(11) $${\hat{x}}_{g}=\frac{\int_{-\infty }^{\infty }\mathrm{xg}(x)\mathrm{dx}}{\int_{-\infty }^{\infty }g(x)\mathrm{dx}}=-\frac{G^{\prime }(0)}{2\pi \mathrm{iG}(0)}$$

As described above, *x̂_c_* is the ideal star centroid position and *x̂_g_* is the actual star centroid position with approximation systematic error. The following step, we will begin to analyze the *x̂_g_* influenced by the approximation systematic error and get its theoretical model through frequency domain analysis.

Starlight can be viewed as point light sources, so the starlight signal intensity distribution spread point function is approximated reasonably by the Gaussian function and the 2-D situation function can be written as [2,10,13]

(12) $$f(x,y)=\frac{I_{0}}{{2\pi \sigma_{\mathrm{PSF}}}^{2}}\text{exp}[-\frac{{\left(x-x_{0}\right)}^{2}+{\left(y-y_{0}\right)}^{2}}{{2\sigma_{\mathrm{PSF}}}^{2}}]$$

For just considering the x direction, the 1-D case can be reduced to:

(13) $$f(x)=\frac{I_{0}}{\sqrt{2\pi }\sigma_{\mathrm{PSF}}}\text{exp}[-\frac{{\left(x-x_{0}\right)}^{2}}{2\sigma_{\mathrm{PSF}}{}^{2}}]$$

where *x*_0_ represents the ideal star centroid position equal to *x̂_c_*, and the *σ_PSF_* is the Gaussian width parameter. Through the Equation (13), *f*(*x*) can be expressed by the *f_e_*(*x*) shifted by offset d from the origin, *i.e.*:

(14) $$f(x)=f_{e}(x-d)$$

From the Equation (13), it can find that the d equals to *x*_0_. The Fourier transform of *f*(*x*) is written as:

(15) $$F(s)=\text{exp}(-2\pi \mathrm{ids})F_{e}(s)$$

where the *F_e_*(*s*) is the Fourier transform of *f_e_*(*s*).

From the Equation (11), the approximation systematic error *σ_X̃_g_, x_* can be written by:

(16) $$\sigma_{{\tilde{X}}_{g},x}={\hat{x}}_{g}-x_{0}=-\frac{G^{\prime }(0)}{2\pi \mathrm{iG}(0)}-x_{0}=-\frac{G^{\prime }(0)}{2\pi \mathrm{iG}(0)}-d$$

From Equation (6), the G(s) can be written by *G* (*x* ) = *F*(*s*) × *T*(*s*), according to the form of *t*(*x*) in Equation (7) and sampling frequency *f_s_* = 1/*T*, the G(s) can be given as:

(17) $$\begin{array}{ll} G(s) & =\sum_{n=-\infty }^{\infty }F(s-n/T)=\sum_{n=-\infty }^{\infty }F(s-{\mathrm{nf}}_{s})\\ & =\sum_{n=-\infty }^{\infty }\text{exp}[-2\pi \mathrm{id}(s-{\mathrm{nf}}_{s})]F_{e}(s-{\mathrm{nf}}_{s}) \end{array}$$

Then the derivative of *G* (*s*) is written by:

(18) $$\begin{array}{ll} G^{\prime }(s) & =\sum_{n=-\infty }^{\infty }\left\{-2\pi \mathrm{id}\text{exp}[-2\pi \mathrm{id}(s-{\mathrm{nf}}_{s})]F_{e}(s-{\mathrm{nf}}_{s})\right\}\\ & +\sum_{n=-\infty }^{\infty }\text{exp}[-2\pi \mathrm{id}(s-{\mathrm{nf}}_{s})]F_{e}^{\prime }(s-{\mathrm{nf}}_{s})\\ & =-2\pi \mathrm{id}*G(s)+\sum_{n=-\infty }^{\infty }\text{exp}[-2\pi \mathrm{id}(s-{\mathrm{nf}}_{s})]F_{e}^{\prime }(s-{\mathrm{nf}}_{s}) \end{array}$$

Then substituting Equations (17) and (18) into (16) yields:

(19) $$\begin{array}{ll} \sigma_{{\tilde{X}}_{g},x} & =\frac{G^{\prime }(0)}{2\pi \mathrm{iG}(0)}-d\\ & =-\frac{-2\pi \mathrm{idG}(0)+\sum_{n=-\infty }^{\infty }{\text{exp}[-2\pi \mathrm{id}(s-{\mathrm{nf}}_{s})]F_{e}^{\prime }(s-{\mathrm{nf}}_{s})|}_{s=0}}{2\pi \mathrm{iG}(0)}-d\\ & =\frac{-\sum_{n=-\infty }^{\infty }\text{exp}[-2\pi \mathrm{id}(s-{\mathrm{nf}}_{s})]F_{e}^{\prime }(s-{\mathrm{nf}}_{s}){|}_{s=0}}{2\pi \mathrm{iG}(0)} \end{array}$$

Substituting s = 0 into Equation (19), and noting that *F_e_*(*x*) is even, and the *F*′*_e_*(*x*) is odd. Then the numerator of the *σ*_*X̃*_*g*_*, x*_ in Equation (19) can be calculated as:

(20) $$\begin{array}{l} -\sum_{n=-\infty }^{\infty }{\text{exp}[-2\pi \mathrm{id}(s-{\mathrm{nf}}_{s})]F_{e}^{\prime }(s-{\mathrm{nf}}_{s})|}_{s=0}\\ =-F_{e}^{\prime }(0)-\sum_{n=1}^{\infty }F_{e}^{\prime }(-{\mathrm{nf}}_{s})[\text{exp}(2\pi \mathrm{id}{\mathrm{nf}}_{s})-\text{exp}(-2\pi \mathrm{id}{\mathrm{nf}}_{s})]\\ =\sum_{n=1}^{\infty }2iF_{e}^{\prime }({\mathrm{nf}}_{s})\text{sin}(2\pi d{\mathrm{nf}}_{s}) \end{array}$$

From Equation (17), the denominator of *σ*_*X̃*_*g*_*, x*_ can be obtained as:

(21) $$\begin{array}{ll} G(0) & =\sum_{n=-\infty }^{\infty }\text{exp}[-2\pi \mathrm{id}(s-{\mathrm{nf}}_{s})]F_{e}(s-{\mathrm{nf}}_{s}){|}_{s=0}\\ & =F_{e}(0)+\sum_{n=1}^{\infty }\left[\text{exp}(2\pi \mathrm{id}{\mathrm{nf}}_{s})+\text{exp}(-2\pi \mathrm{id}{\mathrm{nf}}_{s})]F_{e}(-{\mathrm{nf}}_{s}\right)\\ & =F_{e}(0)+\sum_{n=1}^{\infty }2\text{cos}(2\pi d{\mathrm{nf}}_{s})F_{e}({\mathrm{nf}}_{s}) \end{array}$$

Taking the Equations (20) and (21) into the Equation (19) to get the approximation systematic error *σ*_*X̃*_*g*_*, x*_ as:

(22) $$\begin{array}{ll} \sigma_{{\tilde{X}}_{g},x} & =\frac{2i\sum_{n=1}^{\infty }F_{e}^{\prime }({\mathrm{nf}}_{s})\text{sin}(2\pi d{\mathrm{nf}}_{s})}{2\pi i[F_{e}(0)+\sum_{n=1}^{\infty }2\text{cos}(2\pi d{\mathrm{nf}}_{s})F_{e}({\mathrm{nf}}_{s})]}\\ & =\frac{\sum_{n=1}^{\infty }F_{e}^{\prime }({\mathrm{nf}}_{s})\text{sin}(2\pi d{\mathrm{nf}}_{s})}{\pi [F_{e}(0)+\sum_{n=1}^{\infty }2\text{cos}(2\pi d{\mathrm{nf}}_{s})F_{e}({\mathrm{nf}}_{s})]} \end{array}$$

*f_s_* = 1/*T* is the sampling frequency and we measure all distances in units of the pixel length (*T* = 1), and in Equation (14) the d equals to *x*_0_, so the Equation (22) can be rewritten by:

(23) $$\sigma_{{\tilde{X}}_{g},x}=\frac{\sum_{n=1}^{\infty }F_{e}^{\prime }(n)\text{sin}(2\pi nx_{0})}{\pi [F_{e}(0)+\sum_{n=1}^{\infty }2\text{cos}(2\pi nx_{0})F_{e}(n)]}$$

From Equation (6), it follows that:

(24) $$\begin{array}{ll} F_{e}(s) & =ℱ\{f_{e}(x)\}=ℱ\{I(x,0)⊗\mathrm{rect}(x)\}\\ & =I_{0}\text{exp}[-2{\left(\pi s\sigma_{\mathrm{PSF}}\right)}^{2}]\text{sin}(\pi s)/(\pi s) \end{array}$$

(25) $$F_{e}^{\prime }(s)=I_{0}\text{exp}[-2{\left(\pi s\sigma_{\mathrm{PSF}}\right)}^{2}]\text{cos}(\pi s)/s$$

where ℱ{} denotes the Fourier transform operation. Therefore:

(26) $$\begin{array}{l} F_{e}(0)=I_{0},F_{e}(n)=0\;(n\geq 1)\\ F_{e}^{\prime }(n)={\left(-1\right)}^{n}I_{0}\text{exp}[-2{\left(\pi n\sigma_{\mathrm{PSF}}\right)}^{2}]/n \end{array}$$

Substituting Equation (26) into Equation (23) yields:

(27) $$\sigma_{{\tilde{X}}_{g},x}=\frac{1}{\pi }\sum_{n=1}^{\infty }{\left(-1\right)}^{n}\text{exp}[-2{\left(\pi n\sigma_{\mathrm{PSF}}\right)}^{2}]\text{sin}(2\pi nx_{0})/n$$

Equation (27) is the theory expression of the approximation systematic error of star image centroid estimation with Gaussian distribution shape. Under the fixed sampling frequency (*f_s_* = 1), it can be seen that the approximation error *σ*_*X̃*_*g*_*, x*_ is related with Gaussian width *σ_PSF_* and the ideal star centroid position *x*_0_ and it decreases as the Gaussian width increases. Under the condition of *σ_PSF_* > 0.3, Equation (27) can be written by:

(28) $$\sigma_{{\tilde{X}}_{g},x}=-\frac{1}{\pi }\text{exp}[-2{\left(\pi \sigma_{\mathrm{PSF}}\right)}^{2}]\text{sin}(2\pi x_{0})$$

From the Equation (28), it is seen that there is an approximately sinusoidal relationship between *σ*_*X̃*_*g*_*, x*_ and *x*_0_. The amplitude of *σ*_*X̃*_*g*_*, x*_ decreases as the Gaussian width *σ_PSF_* increases. In the following part, we also use numerical simulations to verify the theoretical expression of the approximate systematic error in Equation (28).

Designing the numerical simulations, the ideal star centroid position *x*_0_ is varied from 0 to 1 with the interval of 0.002, and set the Gaussian width *σ_PSF_* from 0.1 to 1.2 with the interval of 0.1. Because the starlight signal intensity point spread function (PSF) is reasonable approximated by the 2-D Gaussian function in Equation (12) and is symmetrical in the x and y direction. Just the 1-D situation in the x direction is considered. Therefore the actual star centroid position *x̂_g_* can be calculated by the following equation:

(29) $${\hat{x}}_{g}=\frac{\int_{-\infty }^{\infty }\mathrm{xg}(x)dx}{\int_{-\infty }^{\infty }g(x)dx}=\frac{\sum_{i=1}^{n}x_{i}I_{i}}{\sum_{i=1}^{n}I_{i}}$$

Then, the approximation systematic error can be expressed by:

(30) $$\sigma_{{\tilde{X}}_{g},x}={\hat{x}}_{g}-x_{0}$$

There is one premise should be stated. The fill factor of the active pixel sensors is assumed to be 100% and each pixel has the same photon response. Then, the detected signal intensity of the *i-th* pixel is:

(31) $$I_{i}=\int_{x_{i}}^{x_{i}+1}I(x,x_{0})dx$$

where *I*(*x*, *x*_0_) equals to *f*(*x*) in Equation (13).

**Figure 2.**
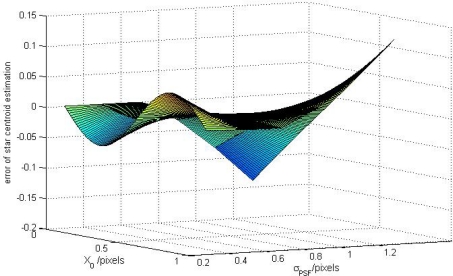
Numerical simulations of the relationship between the approximation systematic error of the COM algorithm and the ideal star centroid positions under different Gaussian widths.

The sampling window size is fixed at 5 × 5 pixels. Under different Gaussian widths, a group of curves between the approximation systematic error *σ*_*X̃*_*g*_, *x*_ and the ideal star centroid position *x*_0_ can be obtained. The 3-D numerical simulation results of the relationships between *σ*_*X̃*_*g*_, *x*_ and *x*_0_ is shown in Figure 2.

Through the Figure 2, it can be seen that the systematic error *σ*_*X̃*_*g*_, *x*_ and the *x*_0_ has an approximately sinusoidal relationship when the Gaussian width *σ_PSF_* is small (*σ_PSF_* < 0.5), and the result is consistent with the theoretical analysis in Equation (28), but, when the *σ_PSF_* is large, there is a linear relationship between *σ*_*X̃*_*g*_, *x*_ and *x*_0_. This is an interesting result, and we will introduce another type of systematic error named truncation systematic error here to describe this phenomenon. The approximation systematic error is caused by the sampling frequency limitation and the truncation systematic error is caused by the sampling window limitation. The truncation error will appear when the size of sampling window is smaller than Gaussian width and will be discussed in detail in the next section.

### 2.3. Theoretical Analysis of the Systematic Truncation Error under Sampling Window Limitation

**Figure 3.**
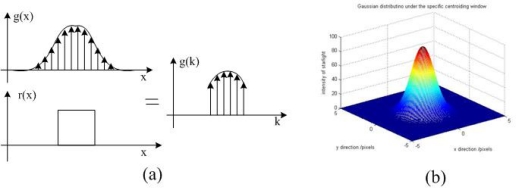
(**a**) The width of Gaussian is larger than the sampling window size; (**b**) The width of Gaussian is smaller than the sampling window size.

In this section, we will analyze the truncation error and give the criterion for choosing the sampling window size to reduce the systematic error as much as possible. The simulations above show that the truncation error will appear when the sampling window size is relatively small. The sampling window area decides how many validated neighbor pixels around the star signal in the image plane were involved in calculating the star centroid position. In Figure 3, we will demonstrate how the sampling window size introduces error into the star centroid position estimation.

Figure 3(a), shows that the Gaussian width *σ_PSF_* is larger than the sampling window size. We can see that the *g*(*k*) is a part of the *g*(*x*) and *g*(*x*) has truncated some effective pixels from the original star signal. Then, the *g*(*k*) has fewer pixels to be used in calculating the star centroid position and will introduce a truncation systematic error to the final star centroid position estimation. In Figure 3(b), the sampling window size is larger than the Gaussian width *σ_PSF_*. In this case, the *g*(*k*) contained all the information of the star signal *g*(*x*) and the size of the sampling window will not cause the truncation systematic error. Under this condition, the error is just dominated by the systematic approximation error.

**Figure 4.**
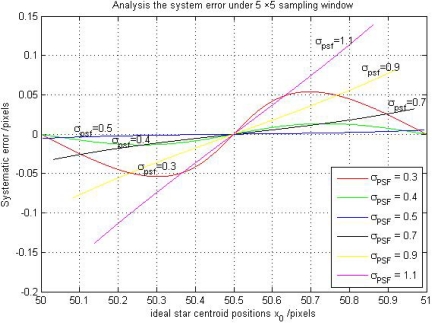
The 2-D result of systematic error of star centroid position estimation under different Gaussian widths.

**Figure 5.**
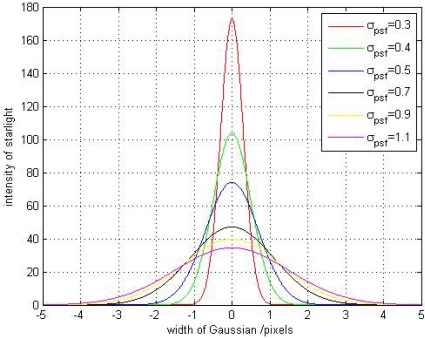
The number of pixels occupied of star under different Gaussian width *σ_PSF_*.

Here, we also use the numerical simulations (designed in Section 2.2) to analyze the truncation systematic error. We select some Gaussian width from 0.1 to 1.2 to implement the simulations again and give the 2-D experiment results under *σ_PSF_* = 0.3, 0.4, 0.5, 0.7, 0.9, 1.1 in Figure 4, and also give out the number of pixels occupied by the Gaussian curve under different Gaussian widths in Figure 5.

From Figure 4, it can be seen that the relationship between *σ*_*X̃*_*g*_, *x*_ and *x*_0_ changed from approximately sinusoidal to linear with the Gaussian width increases. Combining Figures 4 and 5, we can explain the reason of the truncation error clearly. When the Gaussian width is smaller than 0.5, we can find that the number of pixels occupied by the Gaussian curve in Figure 5 is smaller than the 5-pixel window size (the sampling window size selected in our numerical simulations). In this case, the systematic error is just caused by the sampling frequency limitation and is dominated by the approximation error. When the Gaussian width is larger than 0.5, the number of pixels occupied by the Gaussian curve exceeds the 5-pixel window size. In this case, the star signal is truncated by the smaller sampling window size. Only partial effective pixels can be involved in calculating the star centroid position estimation. Under this condition, the error is dominated by the truncation systematic error.

In order to reduce the truncation error as much as possible, a criterion for choosing the size of the sampling window is put forth. The size of sampling window should be a little larger than the Gaussian width. The Gaussian width (PSF size) is decided by the defocusing. If a small displacement *δ_Z_* from the image plane, the Gaussian width will increase and its diameter is [14]:

(32) $$D=\frac{\delta_{Z}}{F\#}$$

where the F# is the optics number of the image sensor. The unit of the D is μm.

The size of sampling window can be chosen following the function below:

(33) $$W_{\mathrm{size}}=\mathrm{fix}(\frac{D}{{\mathrm{Pixel}}_{\mathrm{size}}})+1$$

where fix is the corresponding function in MATLAB, which rounds the elements towards zero. The term *pixel_size_* is the single pixel size of the image plane (e.g., STAR250 *pixel_size_* = 25 *μm*), *D/ pixel_size_* is the Gaussian width of the star signal. In order to let the sampling window size be larger than the Gaussian width, the sampling window size *W_size_* adds one additional pixel on the Gaussian width. Under this operation, we can reduce the truncation systematic error as much as possible. Then, the systematic error of the COM algorithm is just dominated by the approximation error.

Through an appropriate numerical simulation, we can get the relationship between the systematic error *σ*_*X̃*_*g*_, *x*_, the ideal star centroid position *x*_0_ and the actual star centroid position *x̂_g_* contaminated by the error. From the Equation (30), we can calculate the ideal star centroid position *x*_0_ as follows:

(34) $$x_{0}={\hat{x}}_{g}-\sigma_{{\tilde{X}}_{g},x}$$

## 3. The LSSVR Compensation Algorithm

The relationship between the systematic error *σ*_*X̃*_*g*_, *x*_ and the actual star centroid position *x̂_g_* is the basis of our compensation algorithm. We will design a novel algorithm based on the least squares support vector regression (LSSVR) to estimate the systematic error, which can be used to eliminate the systematic error caused by the nature of the COM algorithm.

### 3.1. The Least Squares Support Vector Regression

The support vector machine (SVM) technique was developed by Vapnik in 1995 [15]. SVM is motivated by statistical learning theory based on the principle of structural risk minimization, shown to be superior to the traditional empirical risk minimization principle employed by traditional neural networks. It can be applied in classification and regression. SVR is used to find out the underlying relationships between input and target output vector, especially for modeling nonlinear relationships. It has been proven to be a powerful method for solving problems in nonlinear density estimation and function estimation [16,17]. LSSVR, proposed by Suykens, is an alternate formulation of SVR [18]. The reason for choosing LSSVR as the function estimation is its lower memory requirements, as well as the achievement of a global solution within a fast training speed [19,20]. The primary ridge regression model of LSSVR in the function estimation problem is formulated as:

(35) $$\underset{W,b,\xi }{\text{min}}𝒭(W,b,\xi )=\frac{1}{2}W^{T}W+\gamma \frac{1}{2}\sum_{i=1}^{N}\xi_{i}^{2}$$

subject to the equality constraints:

(36) $$y_{i}=W^{T}\varphi (x_{i})+b+\xi_{i}$$

where *γ* is a positive real constant and *ξ_i_* is slack variable. In this function estimation problem, the Lagrangian is:

(37) $$L_{N}(W,b,\xi ;\alpha )=\frac{1}{2}W^{T}W+\gamma \frac{1}{2}\sum_{i=1}^{N}\xi_{i}^{2}-\sum_{i=1}^{N}\alpha_{i}(W^{T}\varphi (x_{i})+b+\xi_{i}-y_{i})$$

where *α_i_* are Lagrange multipliers. The conditions for optimality are given by [21]:

(38) $$\begin{array}{l} \frac{\partial L_{N}(W,b,\xi ;\alpha )}{\partial_{W}}=0\to W=\sum_{i=1}^{N}\alpha_{i}\varphi (x_{i})\\ \frac{\partial L_{N}(W,b,\xi ;\alpha )}{\partial_{b}}=0\to \sum_{i=1}^{N}\alpha_{i}=0\\ \frac{\partial L_{N}(W,b,\xi ;\alpha )}{\partial_{\xi_{i}}}=0\to \alpha_{i}=\gamma \xi_{i}\\ \frac{\partial L_{N}(W,b,\xi ;\alpha )}{\partial_{\alpha_{i}}}=0\to W^{T}\varphi (x_{i})+b+\xi_{i}-y_{i}=0 \end{array}$$

After eliminating the *W* and *ξ*, the Karush-Kuhn-Tucker (KKT) system is obtained as:

(39) $$\left[\begin{array}{cc} 0 & I_{n}^{T}\\ I_{n} & K+\gamma^{-1}E \end{array}\right]\left[\begin{array}{c} b\\ \alpha \end{array}\right]=\left[\begin{array}{c} 0\\ y \end{array}\right]$$

where *I_n_* = [1,…,1]*^T^*, *α* = [*α*_1_,…, *α_N_*]*^T^*, *y* = [*y*_1_,…, *y_N_*]*^T^*, *K* = *K*(*x_i_,x_j_*) = *φ*(*x_i_*)*^T^* *φ*(*x_j_*). *K*(.,.) is the kernel function, which can be expressed as the inner product of two vectors in some feature space. There are many Mercer kernel functions *K*(*x*,.*x_i_*) that can be chosen, such as $K(x_{i},x_{j})=\text{tanh}[kx_{j}^{T}x_{i}+\theta ]$ (hyperbolic tangent kernel), $K(x_{i},x_{j})={\left(x_{j}^{T}x_{i}+1\right)}^{d}$ (polynomial kernel) and *K*(*x_i_*,*x_j_*) = exp{–||*x_i_* − *x_j_*||^2^ / *σ*^2^} (the RBF kernel). Finally, for an input x, we can predict the output of the LSSVR model in response to the input x as:

(40) $$f(x)=W^{T}\varphi (x_{i})+b=\sum_{i=1}^{N}\alpha_{i}^{*}K(x_{i},x)+b^{*}$$

where $\alpha_{i}^{*}$ and b^*^ are the optimal solutions of Equation (39).

Through the Equation (40), we can find that the LSSVR just calculates sets of linear Equations rather than solving the dual problem in SVR. Furthermore, if we use the RBF kernel, only two parameters (*γ, σ*) are needed for LSSVR in Equation (39). However, except for the parameters (*γ, σ*) are needed in SVR, the parameter *ξ* also should be considered which is the regression error in the e-insensitive loss function. The advantage of low computation complex of LSSVR makes it suitable for our systematic error compensation algorithm.

### 3.2. LSSVR Calculation

The LSSVR model is used for function estimation. In practice, we can’t get the ideal star centroid position *x*_0_ but can get the actual star centroid position *x̂_g_* calculated by Equation (29). According to the Equation (40), we can use the LSSVR to estimate the functional relationship between the systematic error *σ*_*X̃*_*g*_, *x*_ and the actual star centroid position *x̂_g_*. If we use the RBF kernel, the estimation function can be written as:

(41) $$\sigma (x)=\sum_{i=1}^{N}\alpha_{i}^{*}\text{exp}[-{\left(x_{i}-x\right)}^{2}/(2\sigma^{2})]+b^{*}$$

where the x is the input of actual star centroid position *x̂_g_* in practical operation. $\alpha_{i}^{*}$ and b^*^ are the optimal solutions of Equation (39). Then, when we input the *x̂_g_* into the LSSVR model, it will predict its corresponding output of systematic error *σ*_*X̃*_*g*_, *x*_, and we can use Equation (34) to calculate the ideal star centroid position *x*_0_. Through this operation, we can achieve the aim of eliminating the systematic star centroid position error caused by the nature of the COM algorithm.

## 4. Experimental Results and Analysis

In this section, we design a number of experiments to verify the performance of the systematic error compensation algorithm based on the least square support vector regression. The experiments are prepared in three steps. Firstly, before using the LSSVR for function estimation, we should obtain the input training samples through the numerical simulations. Secondly, some parameters can influence the performance of the LSSVR for function estimation. We should use the cross-validation method to get the optimal value of the parameters to guarantee the fitting and prediction accuracy of the LSSVR model. Thirdly, we use our compensation algorithm in the processing of a simulated star image to judge the performance of our proposed LSSVR systematic error compensation algorithm. All these simulations are carried on MATLAB 7.1 software platform run on a Pentium IV 2.8 GHz processor.

### 4.1. Pre-Process the Training Samples

In order to use the LSSVR for regression the relationship between the ideal star centroid position *x*_0_, the actual star centroid position *x̂_g_* (under the systematic error) and the systematic error *σ*_*X̃*_*g*_, *x*_ under different Gaussian width in Equation (34), we should design a number of numerical simulations to get the relationship function among them. Considering the real image sensor STAR250, its image plane size is 512 × 512 pixels, single pixel size is 25 μm, FOV size is 8° × 8°. The starlight projected onto the image plane can be viewed as point light sources, and the starlight signal intensity spread point function is reasonable approximated by the Gaussian function. Just considering the x direction, it can be expressed by Equation (13). We also assume the fill factor of the active pixel sensors is 100% and each pixel has the same photon response. Then, the detected signal intensity function can be given by Equation (31). As mentioned in Sections 2.2 and 2.3, there are two situations that should be considered. The first one is when the sampling window size is larger than the Gaussian width; in this case the systematic error is dominated by the approximation error. Another is when the Gaussian width is larger than the sampling window size; in this case the systematic error is composed of the approximation error and the truncation error. In actual operation, the Gaussian width is increased as the star light intensity is strengthened, soif we use a set sized sampling window, such as 3 × 3 or 5 × 5 pixels, both situations above will exist. The experiments take full consideration of the two situations above, and we set the sampling window size to be 5 × 5 pixels. The Gaussian width *σ_PSF_* is set to be 0.3 (situation 1) and 0.9 (situation 2), respectively. Other values of *σ_PSF_* also can be simulated using the same method and form the compensation template to eliminate the systematic error under different *σ_PSF_* scenarios.

**Figure 6.**
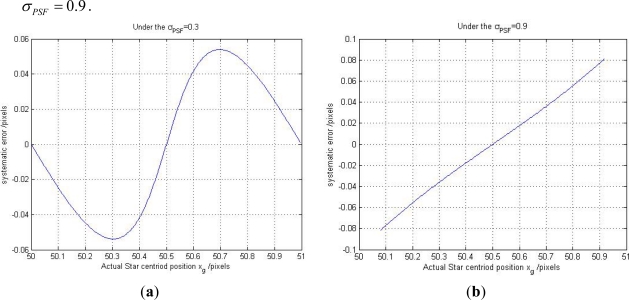
**(a)** The relationship curve between *x̂_g_* and *σ*_*X̃*_*g*_, *x*_ under *σ_PSF_* = 0.3. **(b)** For *σ_PSF_* = 0.9.

We assume the one single starlight is projected on the position (50,160). Just considering the x direction, the star centroid position of the starlight in x direction will range from 50 to 51. We subdivide the one pixel into 300 equivalent parts, and the ideal star centroid position in x direction *x*_0_ from the 50.0033, 50.0066, …, till 51, the simulation step is 0.0033 pixels. If higher star centroid position accuracy is desired, one can reduce the interval of the simulation step but then one must sacrifice the computation time for training the LSSVR. For every trial, we will record the *x*_0_ and the corresponding actual star centroid position *x̂_g_*, then their different is the systematic error *σ*_*X̃*_*g*_, *x*_. Under *σ_PSF_* = 0.3 and 0.9, we can get their relationship, seen in Figure 6.

In Figure 6, we can see that the maximum systematic error is nearly 0.06 pixel under *σ_PSF_* = 0.3 and nearly 0.1 pixel under *σ_PSF_* = 0.9. In the STAR250, one pixel accuracy is 56.25″ Then, 0.06 pixel is approximately 4 arc-second, and the error is big enough to influence the accuracy of the star sensor. It is necessary to design a compensation algorithm to reduce the systematic error. Three hundred training samples can be used to train the LSSVR model to estimate the function above.

### 4.2. The Fitting Accuracy of the LSSVR

**Figure 7.**
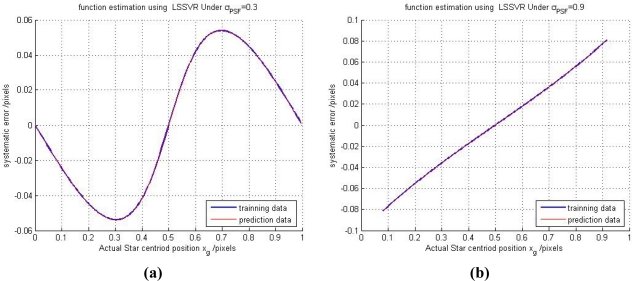
(**a**) The fitting performance of the LSSVR under *σ_PSF_* = 0.3. (**b**) For *σ_PSF_* = 0.9.

The fitting and prediction accuracy are the two main aspects used to judge the quality of our LSSVR model. There are three main parameters that can influence the fitting and prediction accuracy, these are the parameter *σ* of the RBF kernel, degree d of the polynomial function, and parameter *γ* of slack variable in Equation (37). The number of training samples is 300, a relatively small number, so we employ the leave-one-out cross validation approach to choose the optimal parameters. In the optimization of these parameters, the root mean squared error of prediction (RMSEP) of the assessing set is used as an evaluation criterion:

(42) $$\begin{array}{ll} {𝒭}_{\mathrm{RMSEP}} & =\sqrt{\frac{1}{N}\sum_{i=1}^{N}{\left[{\hat{x}}_{g}-Y_{i}-f(x)\right]}^{2}}\\ & =\sqrt{\frac{1}{N}\sum_{i=1}^{N}{\left[{\hat{x}}_{g}-Y_{i}-(\sum_{i=1}^{N}\alpha_{i}^{*}K(x_{i},x)-b^{*})\right]}^{2}}\;\;\;\;\;\;\;i=1\ldots N \end{array}$$

where *Y_i_* is the ideal star centroid position *x*_0_, *f*(*x*) is the prediction output of LSSVR model(with input of actual star centroid position *x̂_g_*). N is the number of prediction samples. Using the criterion of Equation (42), we compared the performance of RBF kernel and the polynomial kernel. The RMSEP of the RBF kernel is smaller than that of polynomial kernel by at least one order of magnitude, so we choose the RBF kernel and the LSSVR parameters *σ* and *γ* are optimized. $\alpha =\sqrt{2}$, *γ* = 2.6 × 10^5^ are used in the calculation. The performance of the regression of the LSSVR is shown in Figure 7.

**Figure 8.**
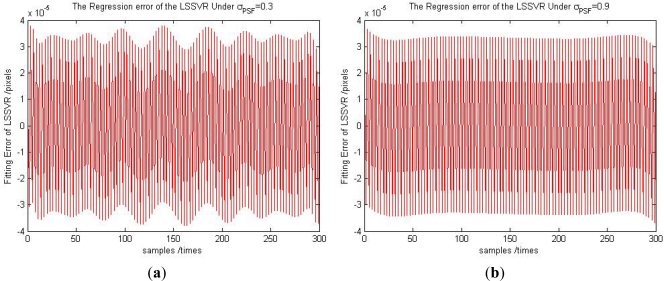
(**a**) The fitting accuracy of the LSSVR under *σ_PSF_* = 0.3. (**b**) For *σ_PSF_* = 0.9.

In Figure 7, we can see that the fitting curve nearly overlaps with the relationship function in Figure 6, and it illustrates that the fitting accuracy of the LSSVR is pretty high under two situations. The corresponding fitting errors of the LSSVR are shown in Figure 8.

The fitting error is defined by the difference between the actual systematic error *σ*_*X̃*_*g*_, *x*_ and the predicted systematic error *σ_lssvr_* which is the output of the LSSVR model. From Figure 8, we can see that under the two situations, the maximum fitting errors of the LSSVR are all smaller than 4 × 10^−5^ pixels, but a high fitting accuracy cannot illustrate the performance of the LSSVR model completely. What we are most concerned with is the prediction accuracy of the LSSVR model.

### 4.3. The Prediction Accuracy of the LSSVR

Firstly, we should give the definition of the prediction accuracy of the LSSVR model. We use the LSSVR model to predict the systematic error *σ_lssvr_* with the input of the actual star centroid position *x̂_g_*, then the star centroid position after compensation can be calculated as:

(43) x˜0=x^g^^−LSSVRpredict(xg)=xg−σlssvr

where *x̂_g_* is the actual star centroid position in practical operation (input of LSSVR), *σ_lssvr_* is the predicted systematic error (output of the LSSVR), and *x̃*_0_ is the star centroid position after compensation. Through the Equation (43), we can get the prediction error of the LSSVR model in the following Equation:

(44) $$\zeta_{\mathrm{predict}}={\tilde{x}}_{0}-x_{0}$$

where *x*_0_ is the ideal star centroid position, *ζ_predict_* is the prediction error of the LSSVR.

**Figure 9.**
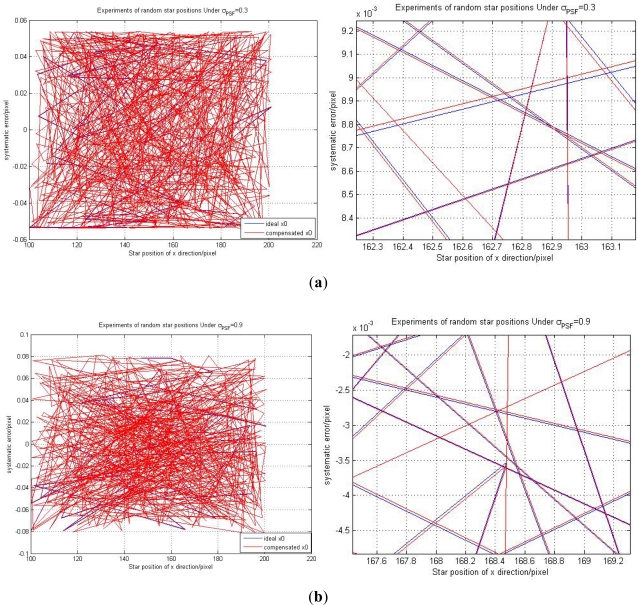
(**a**) Experiments of random star positions under *σ_PSF_* = 0.3. (**b**) For *σ_PSF_* = 0.9.

With the optimal parameters, a LSSVR model was trained using the 300 samples of data in Section 4.1. In order to test the prediction performance of the trained LSSVR model, we select 500 star points which are projected on the CCD image plane randomly. We also just consider the x direction, and all the 500 star centroid positions of the star in the x direction will range from 100 to 201. The experiments are shown in Figure 9.

**Figure 10.**
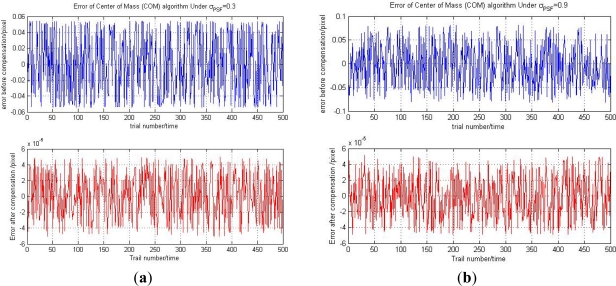
(**a**) The star centroid position error before and after compensation under *σ_PSF_* = 0.3. (**b**) For *σ_PSF_* = 0.9.

The 500 random experiments results under *σ_PSF_* = 0.3 and *σ_PSF_* = 0.9 are shown in the left side of Figure 9(a,b). The right sides of Figure 9(a,b) are corresponding enlarged pictures of the left side. The blue line is the ideal star position *x*_0_ and the red line is the compensated star centroid position *x̃*_0_. From the right side of the Figure 9, we can see that every compensated *x̃*_0_ is very close to its corresponding ideal position *x*_0_. It demonstrates that our trained LSSVR model can achieve high prediction accuracy. The prediction error of the LSSVR model is shown in Figure 10.

From Figure 10, we can see that the prediction errors of our LSSVR model are smaller than 6 × 10^−5^ pixels under the two situations *σ_PSF_* = 0.3 and *σ_PSF_* = 0.9. The result shows that the proposed compensation algorithm can achieve high star centroid position accuracy under different Gaussian widths. The accuracy of our systematic compensation algorithm is much higher than methods proposed by other scholars, such as the neural network method [9] that can reach 5 × 10^−3^ accuracy and the analytical compensation method [10] which can reach 2 × 10^−4^ accuracy.

### 4.4. The Performance of the Compensation Algorithm in Simulations

**Figure 11.**
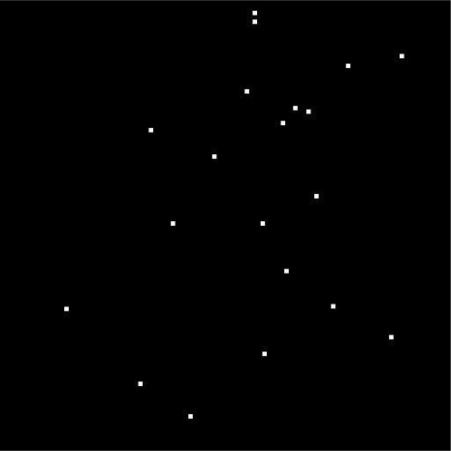
The simulated star image pointing at (130, 60, 60).

In addition to the single star point simulations, we also apply the compensation algorithm to simulated star image testing. We select a star sensor field of view (FOV) point randomly, and suppose the point’s right ascension, declination and the angle rotation are (130, 60, 60). The FOV size is 18 degree, using the sky2000 version 4 star catalog (developed by the NASA’s Goddard Space Flight Center), the stars’ magnitudes in the image are all lower than 6.5 and the *σ_PSF_* = 0.3. The simulated star image is shown in Figure 11.

In Figure 11, we can see that there are 20 stars in the star image. We select 10 of them to compare their errors before compensation and after compensation. The results are shown in Table 1.

Through the experiments above, we can find that the systematic error compensation proposed by the Least Squares Support Vector Regression can achieve high accuracy star centroid positions estimation and meet the high attitude pointing accuracy requirements of star sensors.

### 4.5. The Performance of the Compensation Algorithm in Actual Images Experiments

**Figure 12.**
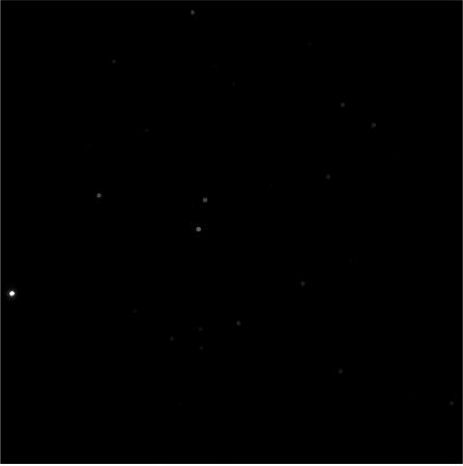
One night sky actual image with FOV 12 × 12 degree.

In addition to the simulated images testing, we also apply the compensation algorithm on some actual images. The actual night sky images were captured on NAOC’s observation station in XingLong, Hebei Province (China), in December 2009. We took about 900 images under different directions. The CANON 20D camera is used, whose focal length is 50 mm, the pixel size is 6.42 μm, the field of view is 25.36 × 17.06 degree, and the plane size is 3,504 × 2,336 pixels. In order to reduce the effects of image distortion, we just used the 12 × 12 degree field of view in the center of each image. One night sky actual image is shown in Figure 12.

We used the zenith observation method to test the accuracy of the star tracker [22,23]. The zenith method takes the Earth as an evenly rotational turntable. It needs a high accuracy spirit level to make sure the star tracker is pointed in the zenith direction. The star tracker captured the pictures from the zenith direction and calculated the attitude. Then, we use our knowledge of astronomy to figure out the ideal zenith direction at the shooting time. Comparing the star tracker’s attitude with the zenith ideal attitude, we can test the accuracy of the star tracker and thus prove the effectiveness of our LSSVR compensation algorithm.

**Figure 13.**
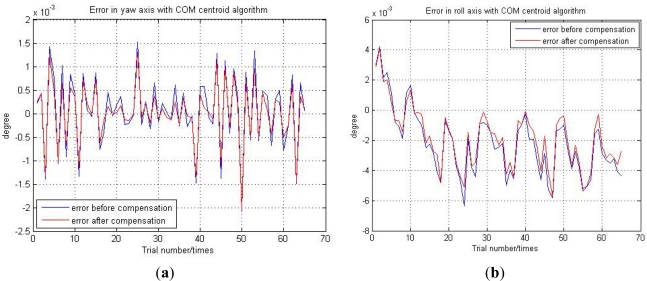
(**a**) The accuracy of the star tracker on the yaw axis. (**b**) The accuracy of the star tracker on the roll axis.

In the 900 sky night actual images, there are about 100 images pointing at the zenith. We selected 66 images to test the accuracy of the star tracker and thus test our LSSVR compensation algorithm. The 66 images are taken under different noise conditions. Through the 66 actual images, we can calculate 66 attitude directions by the star tracker. According to the shooting time and place, we also can calculate 66 ideal zenith directions through the zenith observation method. Before calculating the accuracy of the star tracker, we should eliminate the constant bias on star tracker’s optical axis caused by the assembly. We choose 10 images from the 66 images to calculate the mean of constant bias on the star tracker’s optical axis. After eliminating the constant bias on the optical axis, we can get the accuracy of the star tracker on the yaw axis and roll axis. The experimental results are shown in Figure 13.

From Figure 13, we can see that the accuracy of the star tracker after compensation is higher than before compensation. The actual images experiments can test the performance of our compensation algorithm under different random noise conditions. The 66 actual images are taken under different random noise conditions. Through the Figure 13, we also can see that when the random noise is large, the compensation performance is not obvious. When the random noise is small, the accuracy of the star tracker is very high after compensation. The high performance of our LSSVR compensation algorithm under large random noise condition is to be further studied and improved in our future work.

## 5. Conclusions

This paper analyzed the systematic error of star image centroid estimation utilizing frequency domain analysis and numerical simulations. The sampling frequency limitation and sampling window size limitation are fully considered and the systematic error is then divided into an approximation error and a truncation error. Through the frequency domain analysis, an approximate sinusoidal and linear relationship between systematic error and actual star centroid position are obtained under sampling frequency limitation and under sampling window size limitation, respectively. A novel systematic error compensation algorithm based on the LSSVR is presented. According to the two types of systematic errors, a number of experiments are designed to test the LSSVR compensation algorithm. Simulation results show that after compensation, the residual systematic error of star centroid estimation is less than 6 × 10^−5^ pixels under 5 × 5 pixel sampling window size. Compared to the neural network method and the analytical compensation algorithm, the proposed method’s accuracy is one or two orders of magnitude higher than that of these two algorithms and can meet the requirements of high accuracy star sensors. Since we have not considered the influence of random noise to the proposed method, the high performance of our LSSVR compensation algorithm under large random noise conditions is to be further studied in our future work.

## Figures and Tables

**Table 1.** The systematic error before and after compensation of the simulated star image.

**Star number**	**Ideal x position**	**Actual x position before compensation**	**Error before compensation (pixel)**	**Actual x position after compensation**	**Error after compensation (pixel)**
1	188.533005	188.574817	0.041812	188.5330528	0.0000478
2	33.886746	33.898649	0.011903	33.8867553	0.0000093
3	−83.046154	−83.009843	0.036311	−83.04461024	0.0000516
4	200.032901	199.976565	0.056336	200.0329395	0.0000385
5	94.366492	94.328661	0.037831	94.3664767	0.0000153
6	−38.586794	−38.600159	0.013365	−38.5867838	0.0000102
7	24.180883	24.170196	0.010687	24.1808541	0.0000289
8	79.488555	79.526198	0.037643	79.4885707	0.0000157
9	69.740746	69.773062	0.032316	69.7407809	0.0000349
10	−95.161995	−95.120380	0.041615	−95.1620123	0.0000173
